# Management of sexual dysfunction in breast cancer survivors: a systematic review editorial---looking back to move ahead

**DOI:** 10.1186/s40695-015-0010-y

**Published:** 2015-11-02

**Authors:** G. A. Bachmann, S. Ramachandra

**Affiliations:** Department of Obstetrics, Gynecology and Reproductive Sciences, Rutgers Robert Wood Johnson Medical School, 125 Paterson Street, New Brunswick, 08901 New Jersey USA

This comprehensive review of sexual dysfunction in breast cancer survivors (BCS’s) by Su et al., is a must read for all clinicians who care for not only midlife women who have had breast cancer, but for all women who have had a breast cancer diagnosis, regardless of their age. From these data, although the authors are able to offer useful management strategies, it is clear that there are many opportunities for evaluating current options and exploring future ones for BCS’s with sexual dysfunction. As the authors note, there is still a ‘clinical trials’ road ahead of us that remains to be tread if we are toidentify effective interventions supported by high quality evidence that ultimately will assist BCS’s with their sexual issues and improve their sexual interest, maintain their orgasmic ability and ameliorate or eliminate their genitopelvic pain. What was particularly striking about this extensive literature review was the authors’ recommendation that educational and counseling interventions be included in both the clinical management of the BCS with sexual dysfunction and in clinical trials evaluating prescription and OTC pharmacologic interventions aimed to improve or eliminate sexual dysfunction.

The recommendation of face-time with the patient reinforces much of the ground breaking work done by pioneers in the field of sexual medicine---namely, that sexual dysfunction is multidimensional in origin and that each woman will present with unique circumstances and needs that will demand different approaches and at times multifaceted treatment strategies. Let’s take a short journey back over the past few decades with some examples that reinforce what this review by Su et. al suggest---that education and counseling strategies be provided to the BCS and be included in clinical trials. The reason one-on-one/group education/counseling interventions with patients is critical in patient care and in the evaluation of the safety and efficacy of management strategies in the research setting can be attributed to the psychological, social, cultural, financial and inter- and intrapersonal relationship issues that impact sexual health and wellness. Focusing on BCS’s, the major areas that will adversely impact their sexual function and should be addressed include the woman’s reaction (and her clinician’s) to the diagnosis of the cancer itself, the body alterations that are necessitated by the breast cancer treatment and the new role of ‘patient’ that the otherwise healthy women is suddenly catapulted into.

First, the diagnosis of breast cancer and its overall meaning to the woman (and, when there is one, her sexual partner) and her clinician must be addressed when dealing with the patient and in studying a particular pharmacologic intervention. Early sexual health thought leaders clearly spelled out that the management of sexual dysfunction, especially in BCS’s must encompass the impact of the cancer on both the patient/couple and, not surprisingly, her treating clinician. Over 30 years ago, the textbook, *Sexual Problems in Medical Practice*, (edited by Harold Lief, MD) contains many of the elements critical to addressing sexual issues in patients, including the patient’s and clinician’s reaction to the cancer diagnosis [1]. As stated in this text, the physician has to keep the woman’s sexual health a priority throughout the breast cancer treatment as well as afterwards and not dismiss this aspect of the patient’s life as unimportant. Levay, Sharpe and Kagle, as referenced in this text, further noted, that cancer in general “…is associated with disfigurement after surgery, long and painful illness and high probability of death. It is not surprising that physicians often give the patient’s sexual life low priority” [2]. Therefore, this aspect of cancer’s meaning to the woman and her clinician should be addressed as a part of any intervention being prescribed or studied for management of sexual dysfunctions. That both the clinician and the BCS have to embrace the same outlook about the BCS’s sexual health----namely, that the maintenance of the woman’s sexual wellness is an important aspect of her health prescription and should not be relegated to the column of being unimportant in the overall management algorithm.

Another aspect addressed many decades ago regarding management of sexual issues in the BCS is for the clinician to be sensitive to the changes in body contour that many women are left with after treatment and to understand how the woman has adjusted to these changes. This aspect of the woman’s overall body image has to be considered and discussed, regardless of whether lumpectomy, lymph node dissection, mastectomy, radiation therapy or pharmacologic treatment has been carried out. Breast shape and size may be dramatically changed or an entire breast may be removed with either reconstruction following this surgery or, in some cases, no reconstruction afterwards. The absent or reconstructed breast not only changes body image, but breast and nipple sensation are usually lost or altered as well. As Huffman noted over 40 years ago, “The surgery most traumatic to women seems to be mastectomy, because it so vastly changes feminine appearance and body image” [3]. Ervine further noted, around this same time period, that the patient’s initial reaction (to breast cancer treatment) is to feel “…mutilated, repulsed, desexed, frightened and depressed” [4]. Currently, although mastectomy is not as prevalent today as it was in the 1960’s when this statement was written in the above text, these same feelings can reflect how BCS’s feel regardless of the type of surgery performed. BCS’s deal not only with loss of a previously healthy breast (or breasts) but also with the fatigue, hair loss and adverse body functions that result from the cancer treatment. Giving these women and their partners the tools that educate them on other sexual scripts to use during treatment and beyond is a counseling imperative and should be included and assessed in clinical trials as well.

In addition to the cancer diagnosis itself and the disfigurement that often results, be it mastectomy or lumpectomy, another issue that will contribute to sexual dysfunction is thrusting the midlife BCS who is otherwise healthy into the patient role. In the textbook, *Sexual Desire Disorders* edited by Leiblum and Rosen and published in 1988, the issue of the devastating effect the role of ‘patient’ can have on the sexual functioning of the patient is discussed [5]. In this book, Bullard writes that patients , “…may feel compelled to adopt the ‘patient’ role and to comply with institutional and societal prescriptions that a person with a serious medical condition is ‘asexual’ ” [6]. Here again the education and counseling comes to the forefront in that clinicians must ask patients how they have reacted to the diagnosis of cancer and then to counter any negative role ‘scripts’ that the BCS may express. Education that sexuality does not stop or is not measured by a medical condition, be it chronic or limited, is mandatory, even though this new role as ‘patient’ for the BCS may now require frequent visits to an ambulatory cancer office or a radiation therapy center or a hospital.

What follows is that all of these issues regarding loss, altered body image and feelings of non-optimal health can lead to anxiety, depression, and anger with resultant secondary sexual dysfunction, such as loss of sexual desire and difficulty with sexual arousal and achieving orgasm. With accompanying hormonal changes that may occur at midlife and that can be exacerbated by some breast cancer therapies, vaginal dryness and dyspareunia also can result. Hormonal issues also are of great importance in women who, as a result of family history undergo genetic testing and then decide to follow a surgical path that reduces their risk. That is, women who are found to be at high risk for breast or ovarian cancer can now choose to undergo prophylactic mastectomy and/or oophorectomy, as Angelina Jolie did. These women also have to deal with many of the same issues that women who are diagnosed with breast cancer deal with---altered body image and/or hormonal issues. Many women who undergo this type of preventative surgery are in midlife and, due to the intervention of oophorectomy, immediately become menopausal.

Yes, although we’ve come a long way, as Su et al. note, in managing sexual dysfunction in BCS’s, we still have a long way to go regarding high quality data that will provide us with the tools to optimally manage the sexual issues that often emerge and contribute to expanding more management options for BCS’s. However, it’s important to not lose sight of the lessons provided to us from several decades ago that future management strategies that are implemented and/or studied should be multifaceted and that pharmacological agents should be paired with comprehensive education and counseling interventions.
